# Characterization of a foxtail mosaic virus vector for gene silencing and analysis of innate immune responses in *Sorghum bicolor*

**DOI:** 10.1111/mpp.13270

**Published:** 2022-09-11

**Authors:** Melissa Bredow, Martha Ibore Natukunda, Bliss M. Beernink, Aline Sartor Chicowski, Maria G. Salas‐Fernandez, Steven A. Whitham

**Affiliations:** ^1^ Department of Plant Pathology, Entomology, and Microbiology Iowa State University Ames Iowa USA; ^2^ Department of Agronomy Iowa State University Ames Iowa USA; ^3^ Present address: Department of Biology Augustana University Sioux Falls South Dakota USA.; ^4^ Present address: Department of Biological Sciences University of Manitoba Winnipeg Manitoba Canada.

**Keywords:** foxtail mosaic virus, plant innate immunity, *Pseudomonas syringae*, receptor‐like cytoplasmic kinase, *Sorghum bicolor*, virus‐induced gene silencing, *Xanthomonas vasicola*

## Abstract

Sorghum is vulnerable to many biotic and abiotic stresses, which cause considerable yield losses globally. Efforts to genetically characterize beneficial sorghum traits, including disease resistance, plant architecture, and tolerance to abiotic stresses, are ongoing. One challenge faced by sorghum researchers is its recalcitrance to transformation, which has slowed gene validation efforts and utilization for cultivar development. Here, we characterize the use of a foxtail mosaic virus (FoMV) vector for virus‐induced gene silencing (VIGS) by targeting two previously tested marker genes: *phytoene desaturase* (*PDS*) and *ubiquitin* (*Ub*). We additionally demonstrate VIGS of a subgroup of receptor‐like cytoplasmic kinases (*RLCK*s) and report the role of these genes as positive regulators of early defence signalling. Silencing of subgroup 8 *RLCK*s also resulted in higher susceptibility to the bacterial pathogens *Pseudomonas syringae* pv. *syringae* (B728a) and *Xanthomonas vasicola* pv*. holcicola,* demonstrating the role of these genes in host defence against bacterial pathogens. Together, this work highlights the utility of FoMV‐induced gene silencing in the characterization of genes mediating defence responses in sorghum. Moreover, FoMV was able to systemically infect six diverse sorghum genotypes with high efficiency at optimal temperatures for sorghum growth and therefore could be extrapolated to study additional traits of economic importance.

## Graphical abstract

This study demonstrates that a foxtail mosaic virus vector induces robust gene silencing in sorghum through targeting of two marker genes and a subgroup of receptor‐like cytoplasmic kinases with roles in bacterial defence.

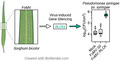

*Sorghum bicolor* (sorghum) is the fifth most economically important cereal crop worldwide (Kumar et al., 2011; Mundia et al., 2019; Rooney et al., 2007). Sorghum is relatively heat‐ and drought‐tolerant compared to other major crops and is cultivated for food, fodder, and biofuel production in many arid and semi‐arid regions (Dillon et al., 2007; Kumar et al., 2011). Currently, more than 90% of the world's sorghum production area is in Africa, Central America, and South Asia, but rising global temperatures are expected to expand cultivation to more northern latitudes (Chadalavada et al., 2021). A major challenge to sorghum production is its vulnerability to a wide range of bacterial, fungal, and viral pathogens (Butsenko & Reshetnikov, 2022; Little & Perumal, 2019; Sharma et al., 2015). Disease management strategies have largely relied on leveraging natural variation in disease resistance between sorghum genotypes (Das, 2019; Mofokeng et al., 2017). Although effective at mitigating crop losses, these strategies can be time‐consuming and often imprecise, resulting in unintended pleiotropic effects or yield penalties (Das, 2019). The most sustainable method to develop elite crops with durable resistance to a broad range of diseases is by identifying and introducing resistance genes using transgenic approaches. There is therefore an urgent need to understand the genetic control of immune signalling networks in this species.

The publication and improved annotation of the sorghum reference genome (Mace et al., 2013; Mullet et al., 2014; Xin et al., 2021) have facilitated the identification of genes regulating traits of interest to sorghum growers. Numerous candidate genes for improved disease resistance have been identified, largely through differential expression analysis using susceptible and resistant genotypes (Cui et al., 2021; Lo et al., 1999; Wang et al., 2020). However, most sorghum genotypes are recalcitrant to standard transformation methods, presenting a major challenge for gene function validation. As a result, the molecular mechanisms that regulate immune responses are poorly understood compared to other staple crops. Virus‐induced gene silencing (VIGS) vectors have been developed as an alternative approach to study gene function in numerous cereal crops, including barley stripe mosaic virus (BSMV) (Hein et al., 2005; Lee et al., 2012), brome mosaic virus (BMV) (Ding et al., 2006; Martin et al., 2011; Scofield & Nelson, 2009), rice tungro bacilliform virus (RTBV) (Kant et al., 2015), foxtail mosaic virus (FoMV) (Liu et al., 2016; Mei et al., 2016), cucumber mosaic virus (CMV) (Wang et al., 2016), and Chinese wheat mosaic virus (CWMV) (Yang et al., 2018). A BMV vector has been characterized for VIGS in sorghum through silencing of the endogenous marker genes *phytoene desaturase* (*PDS*), *ubiquitin* (*Ub*), and *chelatase subunit H* (*ChIH*) (Singh et al., 2018; Singh & Mysore, 2022). BMV‐induced gene silencing has also been used to characterize genes that provide resistance to the fungal pathogens *Colletotrichum sublineolum* and *Setosphaeria turcica* (Biruma et al., 2012; Martin et al., 2011). However, efficient BMV‐induced gene silencing relies on growth at suboptimal temperatures (<22°C) (Singh et al., 2018). Here, we demonstrate the use of FoMV as a robust alternative for VIGS in sorghum and explore its utility for studying innate immune responses through silencing of a subgroup of receptor‐like cytoplasmic kinases (RLCKs) that mediate bacterial immunity.

FoMV is a member of the genus *Potexvirus* of plant RNA viruses and is notable for its wide host range, including 56 Poaceae species (Paulsen, 1977; Short & Davies, 1987). FoMV has a small (c.6.2 kb) monopartite genome composed of five protein‐encoding open reading frames (ORFs). ORF1 encodes an RNA‐dependent RNA polymerase (RdRP) that catalyses replication and transcription from two subgenomic RNAs (Rouleau et al., 1993). ORFs 2–4 encode the triple gene block (TGB) proteins involved in cell‐to‐cell and long‐distance movement (Bruun‐Rasmussen et al., 2008; Samuels et al., 2007). ORF5 encodes the coat protein (CP), which is required for viral encapsidation and aids in viral movement (Cruz et al., 1998; Robertson et al., 2000). Previously, we and others have engineered infectious clones of FoMV capable of transiently silencing endogenous genes in *Zea mays* (maize), *Hordeum vulgare* (barley), *Triticum aestivum* (wheat), *Setaria italica* (foxtail millet), and *Panicum virgatum* (switchgrass) (Liu et al., 2016; Mei et al., 2016; Tiedge et al., 2022). For the purposes of developing a system for gene function validation in sorghum, a viral vector that can infect the wide range of available genotypes is essential. We therefore infected six accessions belonging to the sorghum diversity panel (RTx430, BTx623, PI656015, PI533938, PI533936, and PI533839) (Casa et al., 2008) and assessed systemic infection 21 days postinoculation (dpi) (File S1). Reverse transcription (RT)‐PCR analysis indicated that FoMV was capable of systemically infecting all six genotypes (Figure 1a) with a 100% infection rate. Many viruses have been shown to replicate more efficiently at lower than ambient temperatures, presumably due to reduced antiviral defences (Adelman et al., 2008; Cakir & Tör, 2010; Szittya et al., 2003). FoMV appeared to efficiently replicate in systemic tissues at temperatures optimal for sorghum growth (25–28°C); this is in contrast to BMV, which replicates poorly above 22°C and most efficiently when plants are grown under low temperatures (18°C) prior to infection (Singh et al., 2018). Therefore, FoMV may be more suitable for studying gene function under agriculturally relevant conditions.

**FIGURE 1:**
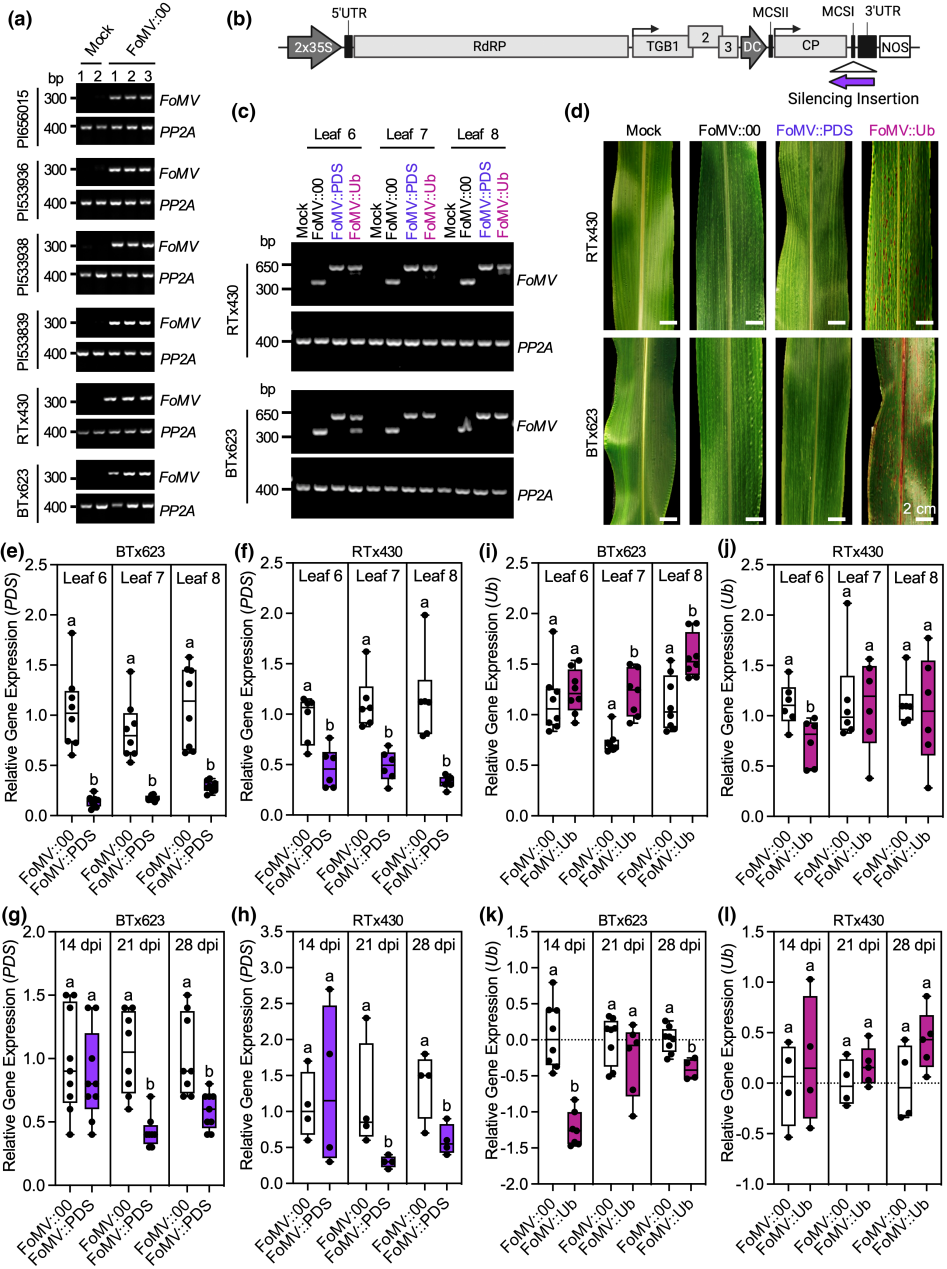
Characterization of FoMV‐mediated gene silencing of marker genes *PDS* and *Ub*. (a) FoMV susceptibility screen using six sorghum genotypes. *FoMV* replication was assessed by reverse transcription (RT)‐PCR analysis at 14 days postinoculation (dpi) in systemic leaf 6. *Protein Phosphatase 2A‐2* (*PP2A*) was used as an internal reference control. (b) FoMV infectious clone used for gene silencing containing five open reading frames (ORFs) encoding an RNA‐dependent RNA polymerase (RdRP), triple gene block movement proteins (TGB1, 2, and 3), and coat protein (CP). The viral genome is flanked by a 2 × 35S cauliflower mosaic virus (CaMV) promoter and a nopaline synthase (NOS) terminator sequence. Gene silencing fragments were cloned into the multiple cloning site (MCSI) in the antisense orientation. (c) Representative RT‐PCR analysis of *PDS* and *Ub* gene fragment stability in RTx430 and BTx623 plants at 21 dpi in systemic leaves 6, 7, and 8. *FoMV* amplicons migrate to 315 bp; *FoMV* amplicons containing *PDS* or *Ub* gene silencing inserts migrate to 625 and 614 bp, respectively. *PP2A* serves as an internal reference control. (d) Representative photographs of viral infection symptoms and *PDS* and *Ub* gene silencing phenotypes in RTx430 and BTx623 plants at 21 dpi (systemic leaf 7). (e, f) RT‐quantitative PCR (RT‐qPCR) analysis of *PDS* gene expression in BTx623 and RTx430 plants at 21 dpi (leaf 7). (g, h) Time‐course analysis in the newest fully expanded leaf at each time point. (i, j) RT‐qPCR analysis of *Ub* gene expression in BTx623 and RTx430 plants at 21 dpi (leaf 7). (k, l) Time‐course analysis in the newest fully expanded leaf at each time point. RT‐qPCR values were calculated relative to *PP2A* expression. Data are represented as box plots indicating the 25%–75% interquartile range, split by a median line. Whiskers represent maximum and minimum values. Statistically significant values (*p* < 0.05) are denoted by different lowercase letters as determined by a one‐way analysis of variance followed by a Tukey's post hoc test using GraphPad Prism 8.0. All experiments were conducted at least three times with similar results.

RTx430 and BTx623 were selected for VIGS experiments because of their well‐annotated genomes and extensive use by the sorghum research community. RTx430 was also particularly interesting to us because of the availability of optimized transformation protocols (Liu & Godwin, 2012) and thus it can be used for gene editing applications (Liu et al., 2019). To generate gene silencing constructs, approximately 300 bp fragments of *PDS* and *Ub* were selected using the Sol Genomics Network VIGS Tool (https://vigs.solgenomics.net/) and cloned in the antisense orientation at the first multiple cloning site (MCSI) of the FoMV genome (Figure 1b, Table S1, and File S1). VIGS of these marker genes in sorghum has been demonstrated using BMV (Singh et al., 2018) and served as a basis for us to assess the relative capacity of FoMV for gene silencing. Retention of gene silencing fragments in the FoMV genome was assessed by RT‐PCR analysis using primers that span the MCSI insertion site (Table S2). At 21 dpi, 72%–90% of BTx623 plants retained *PDS* inserts with at least some fraction of the viral population maintaining the insertion (Figures 1c and S1a). In comparison, 36%–45% of BTx623 plants contained stable *Ub* insertions at the same time point (Figures 1c and S1b). All RTx430 plants retained stable insertions of *PDS* with no obvious deletions while only 12%–25% of RTx430 plants retained stable *Ub* sequences (Figures 1c and S2). Time‐course analysis indicated similar levels of *PDS* retention in both BTx623 and RTx430 plants at 14, 21, and 28 days postinoculation (dpi) (Figures S3a and S4a). In contrast, *Ub* insertions were increasingly unstable at 21 and 28 dpi in both BTx623 and RTx430 plants (Figures S3b and S4b). Together, these results indicate sequence‐ and host genotype‐specific differences in retention of heterologous sequences in FoMV at MCSI.

Of the two sorghum genotypes, FoMV‐infected RTx430 plants displayed more pronounced mosaic symptoms compared to BTx623 plants, which displayed mild symptoms in c.50% of the infected plants (Figure 1d). Viral symptoms were more prominent in BTx623 plants at 14 dpi but became gradually less visible after 21 dpi. Nevertheless, RT‐PCR analysis confirmed systemic viral infections even after visible leaf symptoms subsided (Figure S3). *PDS* silencing results in a visible photobleaching phenotype associated with disrupted carotenoid biosynthesis in barley, maize, and wheat (Holzberg et al., 2002; Mei et al., 2016; Panwar et al., 2013). Interestingly, no photobleaching was observed in any of the FoMV::PDS‐infected RTx430 or BTx623 plants (Figure 1d). However, at 21 dpi reverse transcription real‐time quantitative PCR (RT‐qPCR) analysis indicated 74%–87% and 51%–72% decreases in *PDS* gene expression in BTx623 and RTx430 plants, respectively, compared to FoMV‐infected controls (Figure 1e,f). There was no effect on *PDS* gene expression in FoMV‐treated plants compared to mock‐treated controls (Figure S5). *PDS* gene silencing was observed in the newest fully expanded leaves of BTx623 and RTx430 plants between 21 and 28 dpi, but not at 14 dpi (Figure 1g,h). We additionally observed a 78%–98% reduction in *PDS* gene expression in PI656015, PI533936, PI533839, and PI533938 plants infected with FoMV::PDS at 21 dpi (Figure S6). The lack of a visual phenotype associated with *PDS* gene silencing in sorghum is in line with previous observations using BMV (Singh et al., 2018), and could be because viral symptoms also appear as yellow spots or stripes on leaves. Alternatively, the lack of photobleaching could be due to the presence of an additional homologue of *PDS* (Sobic.001G480550) (Aregawi et al., 2022), which has low sequence identity to the *PDS* VIGS sequence and is unlikely to be silenced.

*Ub* has been demonstrated as an alternative visual marker for gene silencing in sorghum causing cell death (Singh et al., 2018). Unlike *PDS*, FoMV‐induced *Ub* gene silencing resulted in a strong cell death phenotype in BTx623 (Figure 1d), characterized by the development of reddish‐brown lesions (Singh et al., 2018). Cell death symptoms were also observed in RTx430 plants, although this phenotype was less pronounced. Despite the clear visual phenotypes in systemic leaves, at 21 dpi a significant decrease in *Ub* gene expression was only observed in leaf 6 of RTx430 plants (Figure 1i,j). Similarly, in BTx623 plants, leaves displaying a strong *Ub* phenotype did not reduce gene expression of *Ub* compared to FoMV‐treated plants (Figure 1i). We speculated that capturing *Ub* gene silencing could be sensitive to the timing of sample collections because strong silencing would be associated with high levels of cell death. We therefore conducted a time‐course analysis of *Ub* gene silencing at 14, 21, and 28 dpi. We observed silencing at 14 and 28 dpi in the newest fully expanded leaves of BTx623 plants, compared to FoMV‐infected controls (Figure 1k). However, regardless of the sampling time, we were unable to verify gene silencing in RTx430 plants in this analysis (Figure 1l). Together, these results suggest that although *Ub* offers a clearer visual phenotype than *PDS*, the VIGS sequence is inherently less stable and cell death associated with gene silencing could complicate quantitative analysis using this marker gene.

We next sought to investigate the use of FoMV‐mediated silencing in gene function analysis of immune genes. Plants detect pathogens using a suite of plasma membrane‐localized pattern recognition receptors (PRRs) that bind conserved molecules essential to microbial life, known as microbe‐associated molecular patterns (MAMPs) (Couto & Zipfel, 2016; DeFalco & Zipfel, 2021). Binding of MAMPs to cognate PRRs results in the formation of PRR/co‐receptor complexes and the initiation of intracellular signalling in a process known as pattern‐triggered immunity (PTI) (Chai et al., 2013; Chinchilla et al., 2006; Sun et al., 2013). PTI is the first layer of defence against pathogens and involves a complex orchestration of events culminating in broad‐spectrum resistance. The second layer of defence, known as effector‐triggered immunity (ETI), involves the recognition of microbial effectors that allow pathogens to evade or suppress host PTI responses by intracellular nucleotide‐binding leucine‐rich repeat receptors (NB‐LRRs) (Adachi & Kamoun, 2022; Nguyen et al., 2021). To investigate the utility of FoMV‐mediated gene silencing in gene function analysis associated with PTI, we targeted a subgroup of RLCKs with conserved roles in defence signalling in plants (Liang & Zhou, 2018; Sun & Zhang, 2020).

In *Arabidopsis thaliana* (Arabidopsis), subfamily VII RLCKs (RLCK‐VIIs) BOTRYTIS‐INDUCED KINASE 1 (BIK1) and homologue PBS‐Like 1 (PBL1) are rapidly phosphorylated and activated by multiple PRR complexes to initiate intracellular signalling (Couto & Zipfel, 2016; Liang & Zhou, 2018). BIK1 associates with numerous immune signalling proteins including RESPIRATORY BURST OXIDASE HOMOLOGUE D (RBOHD), cyclic nucleotide gated ion channels CNGC2/CNGC4, and calcium‐dependent protein kinases (CDPKs) (Gonçalves Dias et al., 2022; Sun & Zhang, 2020). *bik1 pbl1* loss‐of‐function plants display weakened defence responses and increased susceptibility to bacterial and fungal pathogens (Lu et al., 2010; Veronese et al., 2006; Zhang et al., 2010). Four RLCK‐VII genes have been identified in *Oryza sativa* (rice) (RLCK57, RLCK107, RLCK118, and RLCK176) that are activated by the PRRs XA21 and CERK1, and are associated with resistance to *Xanthomonas oryzae* pv*. oryzae* (Li et al., 2017; Yamaguchi et al., 2013; Zhou et al., 2016). RLCK‐VII genes regulating defence responses have also been identified in barley (Huesmann et al., 2012), maize (Li et al., 2022), and wheat (Wu et al., 2020). To identify RLCK‐VII genes in sorghum, we curated RLCK‐VII gene sequences from Arabidopsis and rice, and performed a BLAST search against the BTx623 sorghum genome (Phytozome v3.1.1). We identified 103 putative RLCK‐VII genes in sorghum (File S2) and aligned them against Arabidopsis and rice RLCKs using the Clustal Omega Multiple Sequence Alignment Tool (Madeira et al., 2022). Three sorghum genes (Sobic.001G033400.1, Sobic.001G421300.1, and Sobic.009G011700.1) clustered with subgroup 8 RLCK‐VII genes, known to positively regulate immune responses, which we have named *RLCK1*, *RLCK2*, and *RLCK3*, respectively (Figure 2a).

**FIGURE 2:**
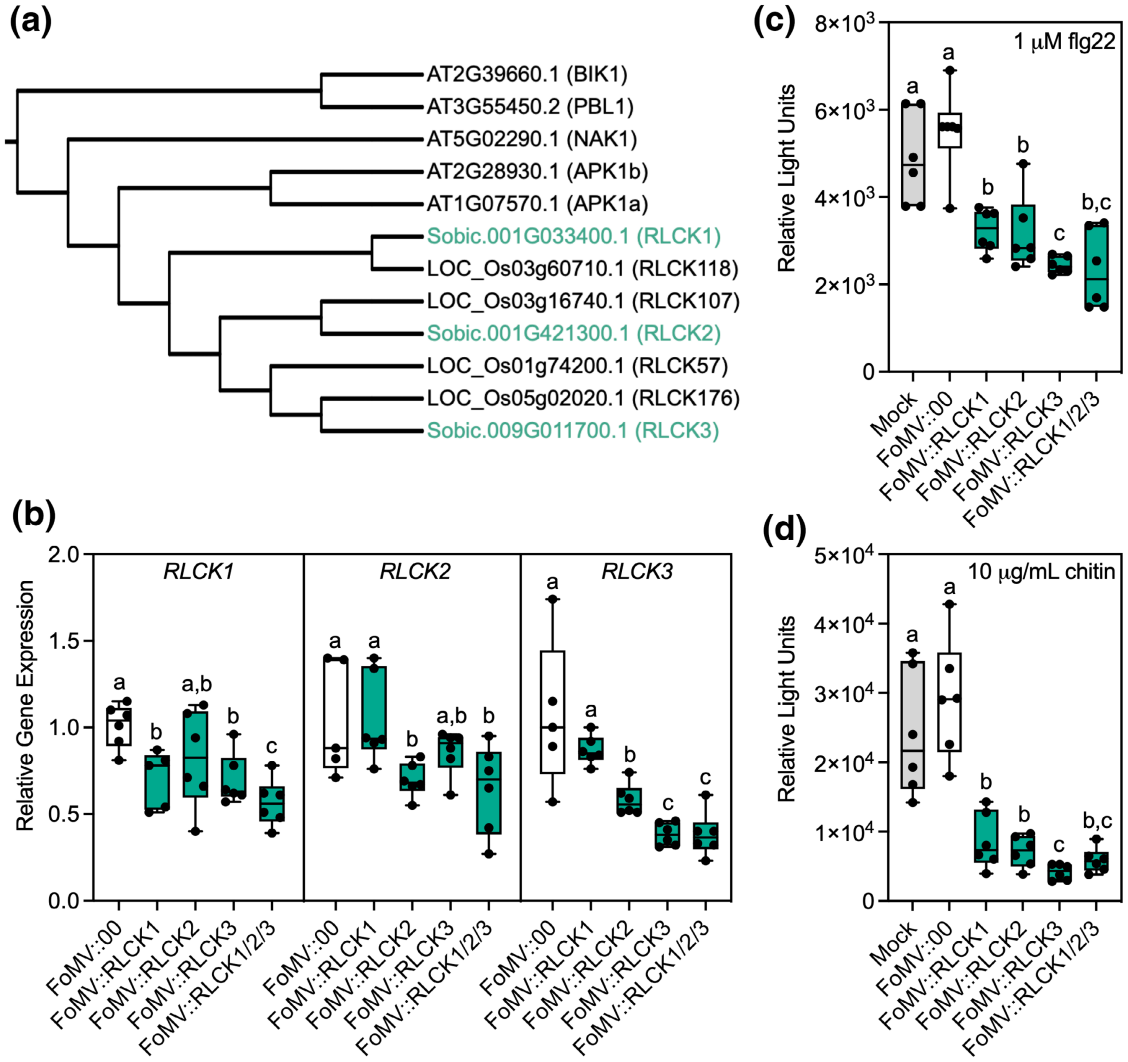
FoMV‐induced gene silencing of subgroup 8 receptor‐like cytoplasmic kinases (RLCKs) in BTx623 sorghum and associated immune‐elicited oxidative species production. (a) Phylogenetic tree of subgroup 8 RLCKs from *Arabidopsis*, rice, and sorghum generated through Clustal Multiple Sequence Alignment (https://www.ebi.ac.uk/Tools/msa/clustalw2/) and reconstructed using the Interactive Tree of Life (iToL) web tool (Ciccarelli et al., 2006). (b) Reverse transcription‐quantitative PCR analysis of *RLCK1*, *RLCK2*, and *RLCK3* VIGS at 21 days postinoculation with FoMV::00, FoMV::RLCK1, FoMV::RLCK2, FoMV::RLCK3, or coinoculated with all three gene silencing constructs (FoMV::RLCK1/2/3). Values were calculated relative to *Protein Phosphatase 2A‐2* (*PP2A*) gene expression. (c) flg22‐ and (d) chitin‐elicited apoplastic reactive oxygen species production in mock, FoMV::00, and FoMV::RLCK1, FoMV::RLCK2, FoMV::RLCK3, and FoMV::RLCK1/2/3 infected plants 21 days after viral infection.

BTx623 was selected for immune assays because of the robust gene silencing we observed with *PDS* and *Ub* (Figure 1) and its susceptibility to bacterial and fungal pathogens (Cui et al., 2021; Patil et al., 2017). Approximately 280–300 bp sequences were selected for VIGS of individual subgroup 8 *RLCKs* (Table S1) using the Sol Genomics Network VIGS Tool (https://vigs.solgenomics.net/) and cloned into FoMV at MCSI in the antisense orientation (Figure 1b). Gene fragments with the least likelihood for off‐target hits were selected by aligning to the sorghum v3.1 reference genome. RT‐PCR analysis indicated that FoMV gene silencing inserts were highly stable in systemic leaves of BTx623 plants at 21 dpi, with the majority of the FoMV population containing full‐length VIGS inserts in all plants (Figure S7). In Arabidopsis BIK1 and PBL1 display partial redundancy in PTI signalling (Zhang et al., 2010); we therefore additionally coinfected plants with FoMV::RLCK1, FoMV::RLCK2, and FoMV::RLCK3 gene silencing constructs. RT‐PCR analysis, using primers designed to differentiate individual VIGS inserts, indicated that five of six plants stably expressed all three VIGS sequences (Figure S8). *RLCK1*, *RLCK2*, and *RLCK3* gene expression was substantially reduced at 21 dpi with individual gene silencing constructs compared to FoMV empty vector (FoMV::00)‐treated plants (Figure 2b). Notably, we did not observe any change in *RLCK* gene expression in FoMV::00 infected plants compared to mock‐treated controls (Figure S9). Given the close sequence homology of these genes (Table S3), we observed some cross‐silencing using FoMV::RLCK2 and FoMV::RLCK3 constructs (Figure 2b), precluding us from investigating the function of these genes individually. Cross‐silencing of closely related genes is a limitation of any VIGS system, and it highlights the need to use additional approaches for further validation. We observed the most robust gene silencing of individual *RLCK* genes when all three FoMV gene silencing vectors were coinfected, which may be associated with silencing of individual RLCKs by multiple VIGS sequences or when infected with FoMV::RLCK3 (Figure 2b).

One of the earliest responses to MAMP detection is the activation of transmembrane RBOH proteins and the production of apoplastic reactive oxygen species (ROS), such as hydrogen peroxide (Kadota et al., 2014; Li et al., 2014). ROS serve as antimicrobial molecules as well as short and long‐distance immune signals from sites of pathogen attack (Lee et al., 2020; Sun & Zhang, 2021). We therefore monitored ROS production in response to the MAMP flg22, corresponding to a 22 amino acid epitope of bacterial flagellin, known to elicit immune signalling in diverse plants including Arabidopsis, rice, soybean, and sorghum (Chinchilla et al., 2006; Cui et al., 2021; Takai et al., 2008; Wei et al., 2020). Silencing of *RLCK1* alone, using the FoMV::RLCK1 silencing vector, significantly reduced flg22‐elicited ROS production compared to FoMV‐treated controls (Figure 2c). The greatest reduction in ROS production was observed using the FoMV::RLCK3 construct or when all three VIGS constructs were coinfected (Figure 2c). Some RLCKs display specificity for PRR complex activation (Rao et al., 2018), therefore we additionally monitored ROS production in response to elicitation with chitin, a component of fungal cell walls and a known MAMP in sorghum (Cui et al., 2021; Nida et al., 2021; Samira et al., 2020). Chitin elicited a more robust ROS burst than flg22 (Figure 2c,d), in line with previous observations in this species (Cui et al., 2021). Silencing of *RLCK1* alone resulted in a significant reduction in chitin‐elicited ROS production (Figure 2d). Again, ROS production was lowest in FoMV::RLCK3‐infected plants or plants infected by all three FoMV:RLCK constructs. No differences were observed in flg22‐ or chitin‐elicited ROS production between mock‐treated plants and those infected with FoMV::00 (Figure 2c,d), indicating no obvious confounding effects associated with viral infection using this assay. Together, these results demonstrate that subgroup 8 RLCKs mediate MAMP‐induced ROS production downstream of at least two PRRs in sorghum.

Three major bacterial diseases occur in sorghum: bacterial leaf stripe caused by *Pseudomonas andropogonis*, bacterial streak caused by *X. vasicola* pv*. holcicola*, and bacterial spot caused by *Pseudomonas syringae* pv*. syringae* (B728a) (Anitha et al., 2020). The aetiology of bacterial diseases has received little attention in sorghum and very few mediators of bacterial defence have been identified. To determine if our VIGS system would allow us to discriminate between disease resistance associated with PTI, infection assays were conducted using two of these pathogens (File S1). Plants coinfected with all three *RLCK* gene silencing vectors were used for these assays because they displayed the most robust gene silencing of individual genes. *RLCK‐*silenced plants were spray‐inoculated with *P. syringae* (B728a) 21 days after viral inoculations. Disease severity was determined by bacterial counts (colony‐forming units; cfu/cm^2^) at 3 dpi and through visual assessment of disease symptoms at 7 dpi. *RLCK*‐silenced plants were more susceptible to *P. syringae* with a roughly 1.5 log‐fold increase in bacterial proliferation (Figure 3a). No differences in bacterial proliferation were observed between mock‐ and FoMV::00‐treated plants. Moreover, visual symptoms associated with bacterial spot disease, characterized by the appearance of irregular shaped tan lesions with red borders, were noticeably larger and more numerous on *RLCK*‐silenced plants compared to FoMV::00 or mock‐treated controls (Figure 3b). Similarly, these plants were more susceptible to *X. vasicola* pv*. holcicola* (Mex‐1), as demonstrated by increased bacterial counts 24 hours postinfection (hpi) (Figure 3c) and lesion length 6 dpi (Figure 3d,e). Together, our results demonstrate that FoMV‐induced gene silencing can be used as an effective system for the investigation of early and late immune responses, assessed through elicitor‐induced ROS production and pathogen susceptibility, respectively. Moreover, we report the role of subgroup 8 RLCK‐VII genes and the impact of PTI in defence against two bacterial pathogens in this species.

**FIGURE 3:**
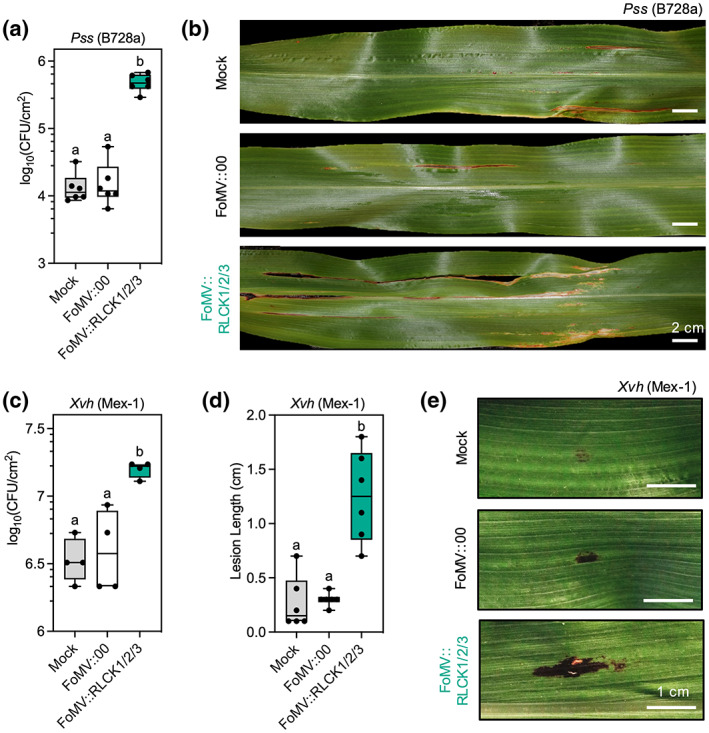
Susceptibility of subgroup 8 *RLCK* VIGS plants to *Pseudomonas syringae* pv*. syringae* (B728a) and *Xanthomonas vasicola* pv. *holcicola* (Mex‐1). (a) Bacterial proliferation counts and (b) infection symptoms from mock, FoMV::00, and FoMV::RLCK1/2/3 treated plants at 3 and 7 days after infection, respectively, with *P. syringae pv. syringae* (B728A) (Pss). (c) Bacterial proliferation counts at 24 h after infiltration with *X. vasicola* pv*. holicola* (Mex‐1) (Xvh). (d) Lesion length measurements and (e) representative photographs taken 6 days after Xvh infiltration. Data are represented as box plots indicating the 25%–75% interquartile range, split by a median line. Whiskers represent maximum and minimum values. Statistically significant values (*p* < 0.05) are denoted by different lowercase letters as determined by a one‐way analysis of variance followed by a Tukey's post hoc test using GraphPad Prism 8.0. All experiments were conducted at least three times with similar results.

The cultivation of sorghum as a global crop has been rapidly expanding, with more than 55 million tonnes of sorghum harvested in 2021 (Shahbandeh, 2022). Anthropogenic climate change is likely to place unforeseen pressures on sorghum production. Changes in temperature and relative humidity could affect susceptibility to plant pathogens that are not presently considered an urgent concern. It is therefore crucial to understand the genetics that underlie responses to both biotic and abiotic stresses. The successful use of FoMV‐induced gene silencing for functional validation of *RLCK*s in this study suggests that its mild infection symptoms and ability to replicate efficiently at physiologically relevant temperatures makes this system a reliable method for validating the function of immune signalling proteins. Moreover, FoMV can silence endogenous gene expression in six distinct sorghum genotypes with high efficiency, highlighting its potential use in gene function validation in other sorghum varieties. Future studies could also utilize FoMV‐mediated overexpression (Bouton et al., 2018; Mei et al., 2019) to complement functional analysis by VIGS. Together, this work provides an efficient alternative to transgenic approaches to study gene function in sorghum, overcoming the current challenges presented by its recalcitrance to transformation.

## Supporting information


**Figure S1** Reverse transcription (RT)‐PCR analysis of (a) *PDS* and (b) *Ub* insert retention in FoMV. Samples were collected from leaves 6–8 of BTx623 sorghum plants at 21 days after inoculation with FoMV::PDS or FoMV::Ub gene silencing constructs. Primers were designed to flank the MCSI cloning site to assess insert retention. RT‐PCR amplification products containing intact *PDS* and *Ub* gene fragments migrate to 625 and 614 bp, respectively. Amplification products derived from FoMV with no MCSI insertion migrate to 315 bp. *Protein Phosphatase 2A‐2* (*PP2A*) was used as a reference control. Experiments were done three times with similar results

**Figure S2** Reverse transcription (RT)‐PCR analysis of (a) *PDS* and (b) *Ub* insert retention in FoMV. Samples were collected from leaves 6–8 of RTx430 sorghum plants at 21 days after inoculation with FoMV::PDS or FoMV::Ub gene silencing constructs. Primers were designed to flank the MCSI cloning site to assess insert retention. RT‐PCR amplification products containing intact *PDS* and *Ub* gene fragments migrate to 625 and 614 bp, respectively. Amplification products derived from FoMV with no MCSI insertion migrate to 315 bp. *Protein Phosphatase 2A‐2* (*PP2A*) was used as a reference control. Experiments were done three times with similar results

**Figure S3** Stability of (a) *PDS* and (b) *Ub* inserts in the FoMV genome at 14, 21, and 28 days postinoculation (dpi) in BTx623 leaves. The newest fully expanded leaf was tested at each time point. Amplicons representing intact *PDS* and *Ub* inserts migrate to 625 and 614 bp, respectively. FoMV empty vector amplicons migrate to 315 bp. *Protein Phosphatase 2A‐2* (*PP2A*) was used as an internal reference control. Experiments were conducted three times with similar results

**Figure S4** Reverse transcription (RT)‐PCR analysis of (a) *PDS* and (b) *Ub* insert retention in the FoMV genome. The newest fully expanded leaves of RTx430 plants were sampled at 14, 21, and 28 days after viral inoculation. Amplification products of intact *PDS* and *Ub* inserts in FoMV migrate to 625 and 614 bp, respectively. FoMV empty vector amplicons migrate to 315 bp. *Protein Phosphatase 2A‐2* (*PP2A*) was used as an internal reference control. Experiments were conducted three times with similar results

**Figure S5** Reverse transcription‐quantitative PCR analysis of *PDS* and *Ub* gene expression in mock and FoMV‐treated (a, b) BTx623 and (c, d) RTx430 plants 21 days postinoculation (leaf 8). Gene expression was normalized to *Protein Phosphatase 2A‐2* (*PP2A*) gene expression. Data are represented as box plots indicating the 25%–75% interquartile range, split by a median line. Whiskers represent maximum and minimum values. Statistically significant values (*p* < 0.05) are denoted by different lowercase letters as determined by a one‐way analysis of variance followed by a Tukey’s post hoc test. All experiments were conducted at least two times with similar results

**Figure S6** Reverse transcription‐quantitative PCR of *PDS* gene expression in (a) P1656015, (b) PI533936, (c) PI533938, and (d) PI533839 sorghum at 21 days postinoculation (leaf 7). *PDS* gene expression was normalized to *Protein Phosphatase 2A‐2* (*PP2A*) gene expression. Data are represented as box plots displaying the 25%–75% interquartile range, split by a median line. Whiskers represent maximum and minimum values. Significantly different values (*p* < 0.05) were determined using a one‐way analysis of variance followed by Tukey’s post hoc test and are represented by different lowercase values. Experiments were conducted three times with similar results

**Figure S7** Reverse transcription‐PCR analysis of *RLCK* gene fragment stability in FoMV. (a) Leaf 6 and (b) leaf 7 were sampled at 21 days postinoculation. Amplicons representing FoMV with no gene inserts migrate to 315 bp. Intact FoMV containing *RLCK1* and *RLCK2* gene fragments migrate to 614 bp, and FoMV containing the *RLCK3* gene fragment migrates to 594 bp. RLCK1/2/3 plants were coinfected with FoMV::RLCK1, FoMV::RLCK2, and FoMV::RLCK3 vectors. *Protein Phosphatase 2A‐2* (*PP2A*) was used as an internal reference control. Experiments were conducted three times with similar results

**Figure S8** Reverse transcription‐PCR analysis of *RLCK1, RLCK2*, and *RLCK3* gene fragment inserts in FoMV in BTx623 plants coinfected with all three *RLCK* gene silencing constructs. An FoMV forward primer was used with gene‐specific reverse primers to differentiate viral constructs (see Table S2). Amplicons for FoMV containing *RLCK1*, *RLCK2*, or *RLCK3* gene fragments migrate to 279, 304, or 282 bp, respectively. *Protein Phosphatase 2A‐2* (*PP2A*) was used as an internal reference control. The experiment was conducted two times with similar results

**Figure S9** Reverse transcription‐quantitative PCR analysis of endogenous *RLCK1*, *RLCK2*, and *RLCK3* gene expression levels in mock and FoMV empty vector‐treated plants. Gene expression was calculated relative to *Protein Phosphatase 2A‐2* (*PP2A*). Data are presented as box plots displaying the 25%–75% interquartile range, split by a median line. Whiskers represent maximum and minimum values. Statistical significance (*p* < 0.05) was determined using a one‐way analysis of variance followed by Tukey’s post hoc test and are denoted by different lowercase letters

**Table S1** Gene fragments used for FoMV‐induced gene silencing in sorghum

**Table S2** Primer sequences used for reverse transcription (RT)‐PCR and RT‐quantitative PCR

**Table S3** Nucleic acid identity of RLCK1, RLCK2, and RLCK3 gene fragments used for FoMV virus‐induced gene silencing

**File S1** Supplemental experimental procedures. Viral vector construction, viral inoculum preparation and sorghum inoculation, FoMV‐induced gene silencing, RT‐PCR and RT‐qPCR analysis, immune assays, supplemental references

**File S2** Putative RLCK‐VII genes in sorghum

## Data Availability

All relevant data are presented in the figures and supporting materials.
