# Supplementary material for: Characterization of a foxtail mosaic virus vector for gene silencing and analysis of innate immune responses in Sorghum bicolor
Source: Mol Plant Pathol. 2022 Sep 11;24(1):71–9. doi: 10.1111/mpp.13270 (PMC9742499; doi:10.1111/mpp.13270)
Supplement: Supplementary file 1 — Figure S1 Reverse transcription (RT)‐PCR analysis of (a) PDS and (b) Ub insert retention in FoMV. Samples were collected from leaves 6–8 of BTx623 sorghum plants at 21 days after inoculation with FoMV::PDS or FoMV::Ub gene silencing constructs. Primers were designed to flank the MCSI cloning site to assess insert retention. RT‐PCR amplification products containing intact PDS and Ub gene fragments migrate to 625 and 614 bp, respectively. Amplification products derived from FoMV with no MCSI insertion migrate to 315 bp. Protein Phosphatase 2A‐2 (PP2A) was used as a reference control. Experiments were done three times with similar results [file MPP-24-71-s010.docx]

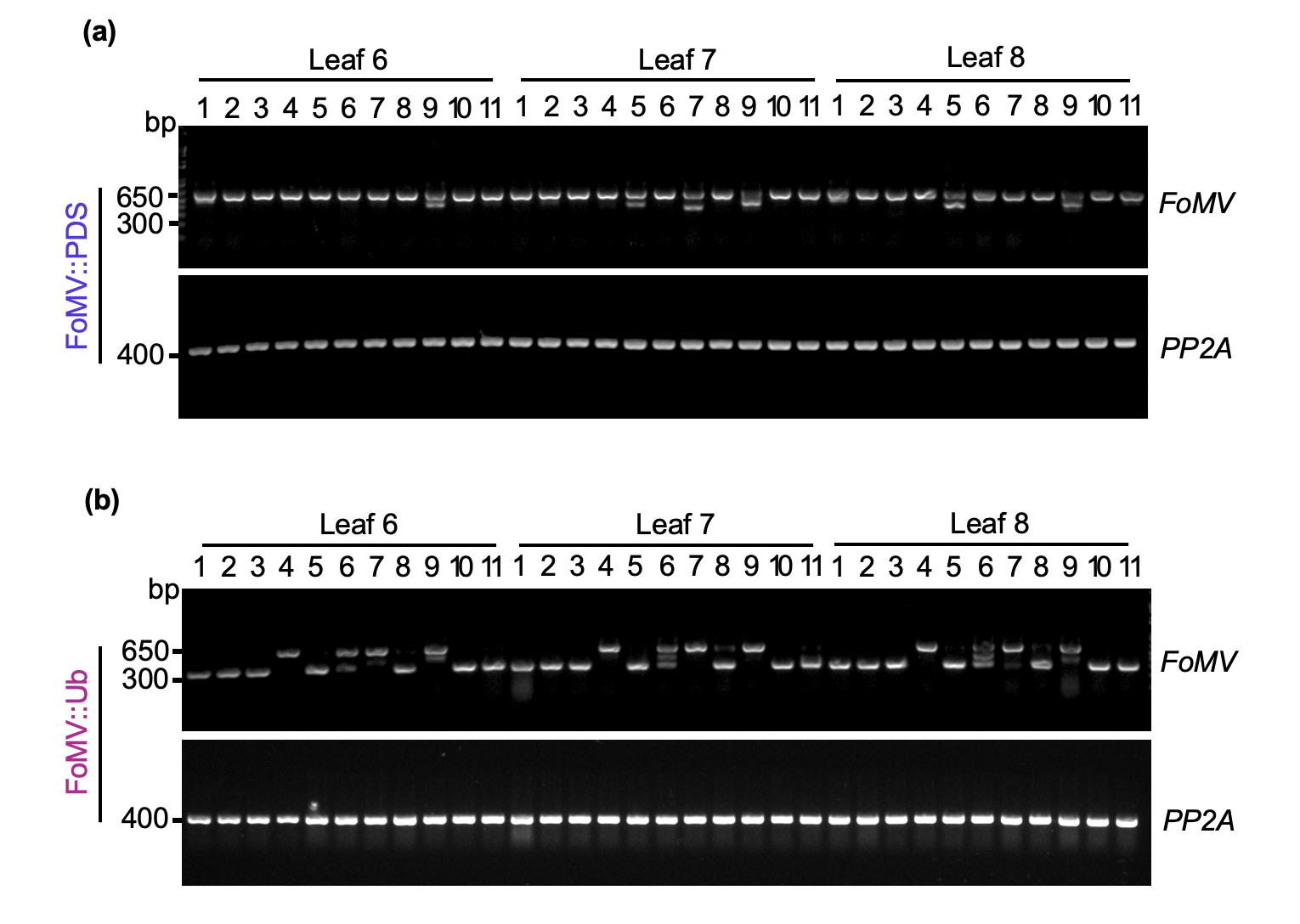


Figure S1. RT-PCR analysis of (a) *PDS* and (b) *Ub* insert retention in FoMV. Samples were collected from leaves 6-8 of BTx623 sorghum plants at 21 days after inoculation with FoMV::PDS or FoMV::Ub gene silencing constructs. Primers were designed to flank the MCSI cloning site in order to assess insert retention. RT-PCR amplification products containing intact *PDS* and *Ub* gene fragments migrate to 625 and 614 bp, respectively. Amplification products derived from FoMV with no MCSI insertion migrate to 315 bp. *Protein Phosphatase 2A-2* (*PP2A*) was used as a reference control. Experiments were done 3 times with similar results.
