# Supplementary material for: Characterization of a foxtail mosaic virus vector for gene silencing and analysis of innate immune responses in Sorghum bicolor
Source: Mol Plant Pathol. 2022 Sep 11;24(1):71–9. doi: 10.1111/mpp.13270 (PMC9742499; doi:10.1111/mpp.13270)
Supplement: Supplementary file 3 — Figure S3 Stability of (a) PDS and (b) Ub inserts in the FoMV genome at 14, 21, and 28 days postinoculation (dpi) in BTx623 leaves. The newest fully expanded leaf was tested at each time point. Amplicons representing intact PDS and Ub inserts migrate to 625 and 614 bp, respectively. FoMV empty vector amplicons migrate to 315 bp. Protein Phosphatase 2A‐2 (PP2A) was used as an internal reference control. Experiments were conducted three times with similar results [file MPP-24-71-s014.docx]

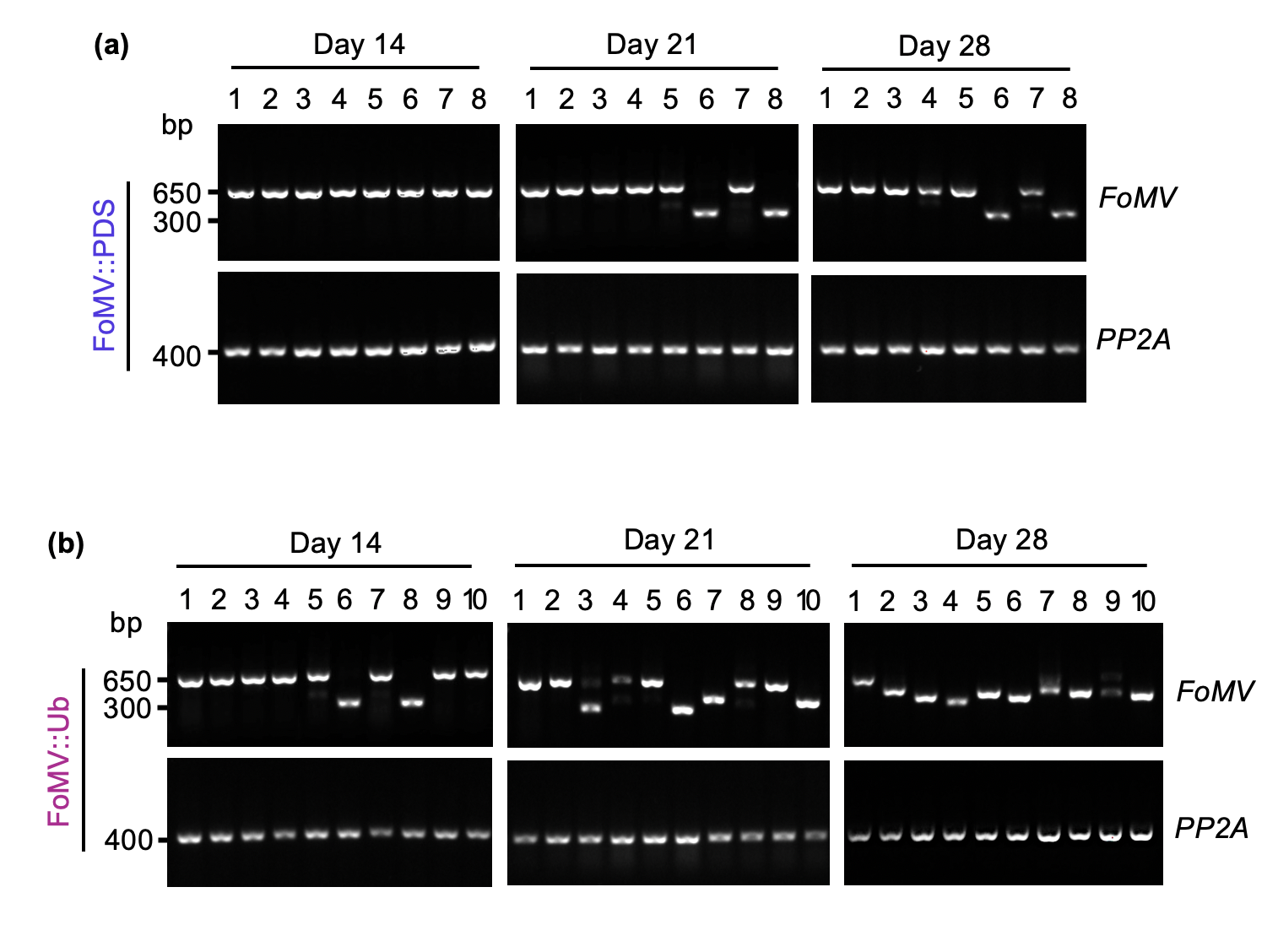


Figure S3. Stability of (a) *PDS* and (b) *Ub* inserts in the FoMV genome at 14, 21, and 28 dpi in BTx623 leaves. The newest fully expanded leaf was tested at each time point. Amplicons representing intact *PDS* and *Ub* inserts migrate to 625 bp and 614 bp, respectively. FoMV empty vector amplicons migrate to 315 bp. *Protein Phosphatase 2A-2* (*PP2A*) was used as an internal reference control. Experiments were conducted three times with similar results.
