# Supplementary material for: Characterization of a foxtail mosaic virus vector for gene silencing and analysis of innate immune responses in Sorghum bicolor
Source: Mol Plant Pathol. 2022 Sep 11;24(1):71–9. doi: 10.1111/mpp.13270 (PMC9742499; doi:10.1111/mpp.13270)
Supplement: Supplementary file 4 — Figure S4 Reverse transcription (RT)‐PCR analysis of (a) PDS and (b) Ub insert retention in the FoMV genome. The newest fully expanded leaves of RTx430 plants were sampled at 14, 21, and 28 days after viral inoculation. Amplification products of intact PDS and Ub inserts in FoMV migrate to 625 and 614 bp, respectively. FoMV empty vector amplicons migrate to 315 bp. Protein Phosphatase 2A‐2 (PP2A) was used as an internal reference control. Experiments were conducted three times with similar results [file MPP-24-71-s002.docx]

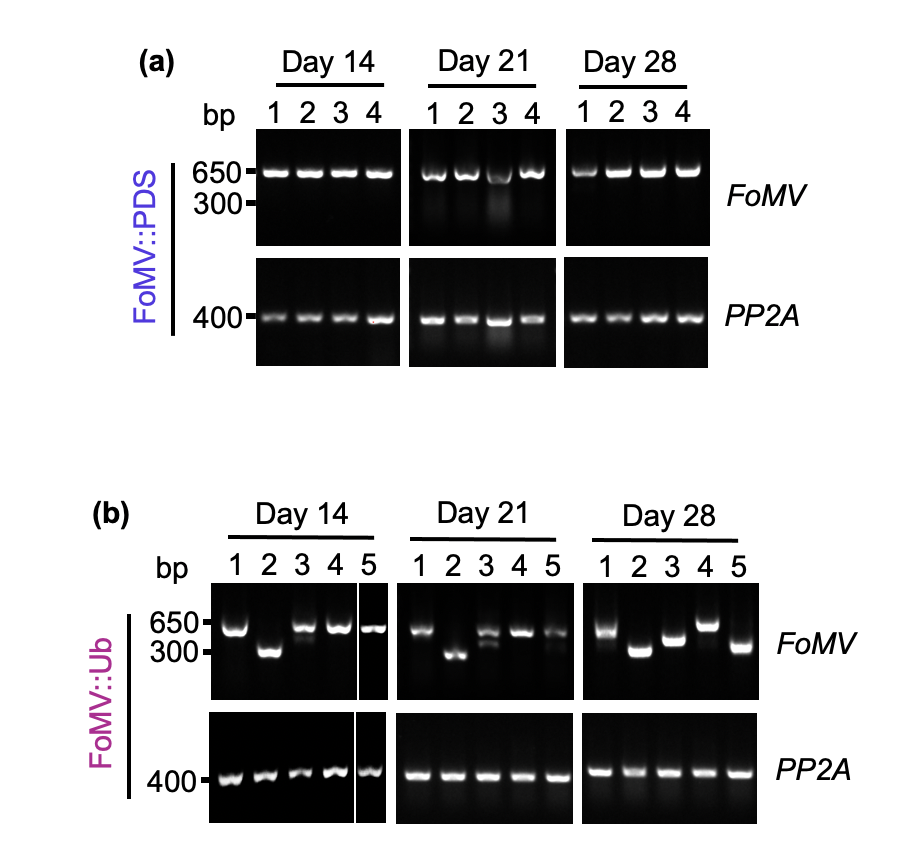


Figure S4. RT-PCR analysis of (a) *PDS* and (b) *Ub* insert retention in the FoMV genome. The newest fully expanded leaves of RTx430 plants were sampled at 14, 21, and 28 days after viral inoculation. Amplification products of intact *PDS* and *Ub* inserts in FoMV migrate to 625 bp and 614 bp, respectively. FoMV empty vector amplicons migrate to 315 bp. *Protein Phosphatase 2A-2* (*PP2A*) was used as an internal reference control. Experiments were conducted three times with similar results. White lines indicate where gel images were merged.
