# Supplementary material for: Characterization of a foxtail mosaic virus vector for gene silencing and analysis of innate immune responses in Sorghum bicolor
Source: Mol Plant Pathol. 2022 Sep 11;24(1):71–9. doi: 10.1111/mpp.13270 (PMC9742499; doi:10.1111/mpp.13270)
Supplement: Supplementary file 5 — Figure S5 Reverse transcription‐quantitative PCR analysis of PDS and Ub gene expression in mock and FoMV‐treated (a, b) BTx623 and (c, d) RTx430 plants 21 days postinoculation (leaf 8). Gene expression was normalized to Protein Phosphatase 2A‐2 (PP2A) gene expression. Data are represented as box plots indicating the 25%–75% interquartile range, split by a median line. Whiskers represent maximum and minimum values. Statistically significant values (p < 0.05) are denoted by different lowercase letters as determined by a one‐way analysis of variance followed by a Tukey’s post hoc test. All experiments were conducted at least two times with similar results [file MPP-24-71-s001.docx]

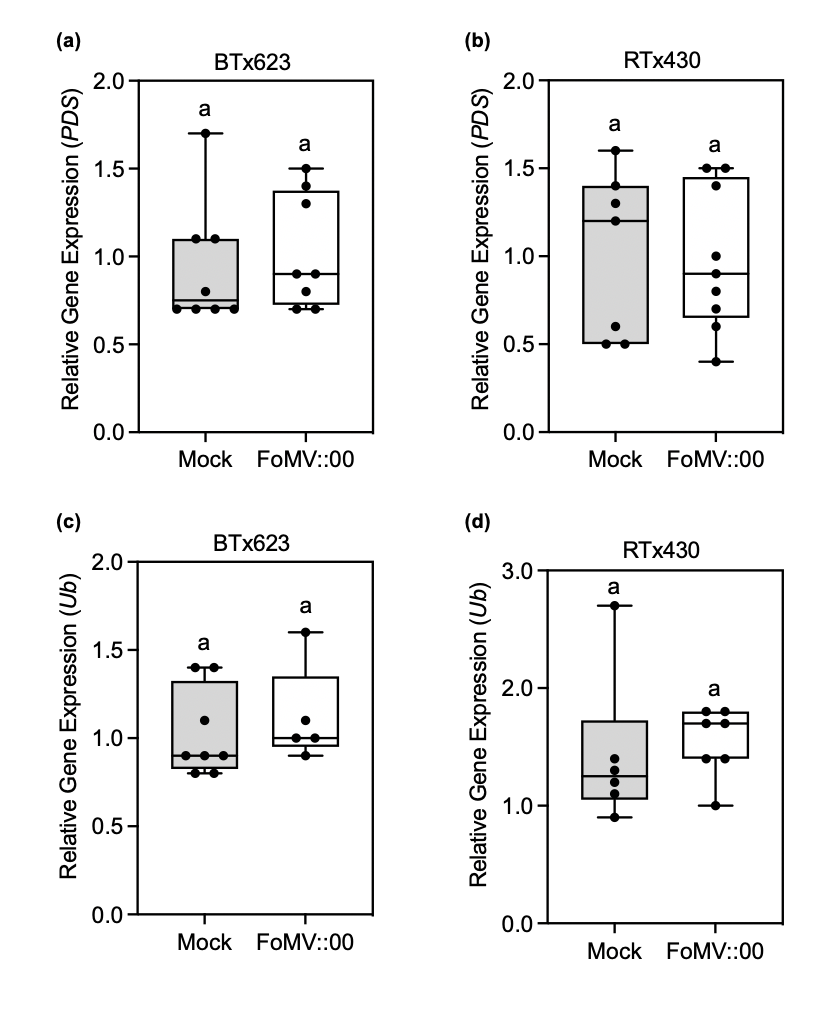


Figure S5. RT-qPCR analysis of *PDS* and *Ub* gene expression in mock and FoMV-treated (a-b) BTx623 and (c-d) RTx430 plants 21 dpi (leaf 8). Gene expression was normalized to *Protein Phosphatase 2A-2* (*PP2A*) gene expression. Data are represented as box plots indicating the 25-75% interquartile range, split by a median line. Whiskers represent maximum and minimum values. Statistically significant values (p<0.05) are denoted by different lowercase letters as determined by a one-way ANOVA followed by a Tukey’s post hoc test. All experiments were conducted at least two times with similar results.
