# Supplementary material for: Characterization of a foxtail mosaic virus vector for gene silencing and analysis of innate immune responses in Sorghum bicolor
Source: Mol Plant Pathol. 2022 Sep 11;24(1):71–9. doi: 10.1111/mpp.13270 (PMC9742499; doi:10.1111/mpp.13270)
Supplement: Supplementary file 6 — Figure S6 Reverse transcription‐quantitative PCR of PDS gene expression in (a) P1656015, (b) PI533936, (c) PI533938, and (d) PI533839 sorghum at 21 days postinoculation (leaf 7). PDS gene expression was normalized to Protein Phosphatase 2A‐2 (PP2A) gene expression. Data are represented as box plots displaying the 25%–75% interquartile range, split by a median line. Whiskers represent maximum and minimum values. Significantly different values (p < 0.05) were determined using a one‐way analysis of variance followed by Tukey’s post hoc test and are represented by different lowercase values. Experiments were conducted three times with similar results [file MPP-24-71-s006.docx]

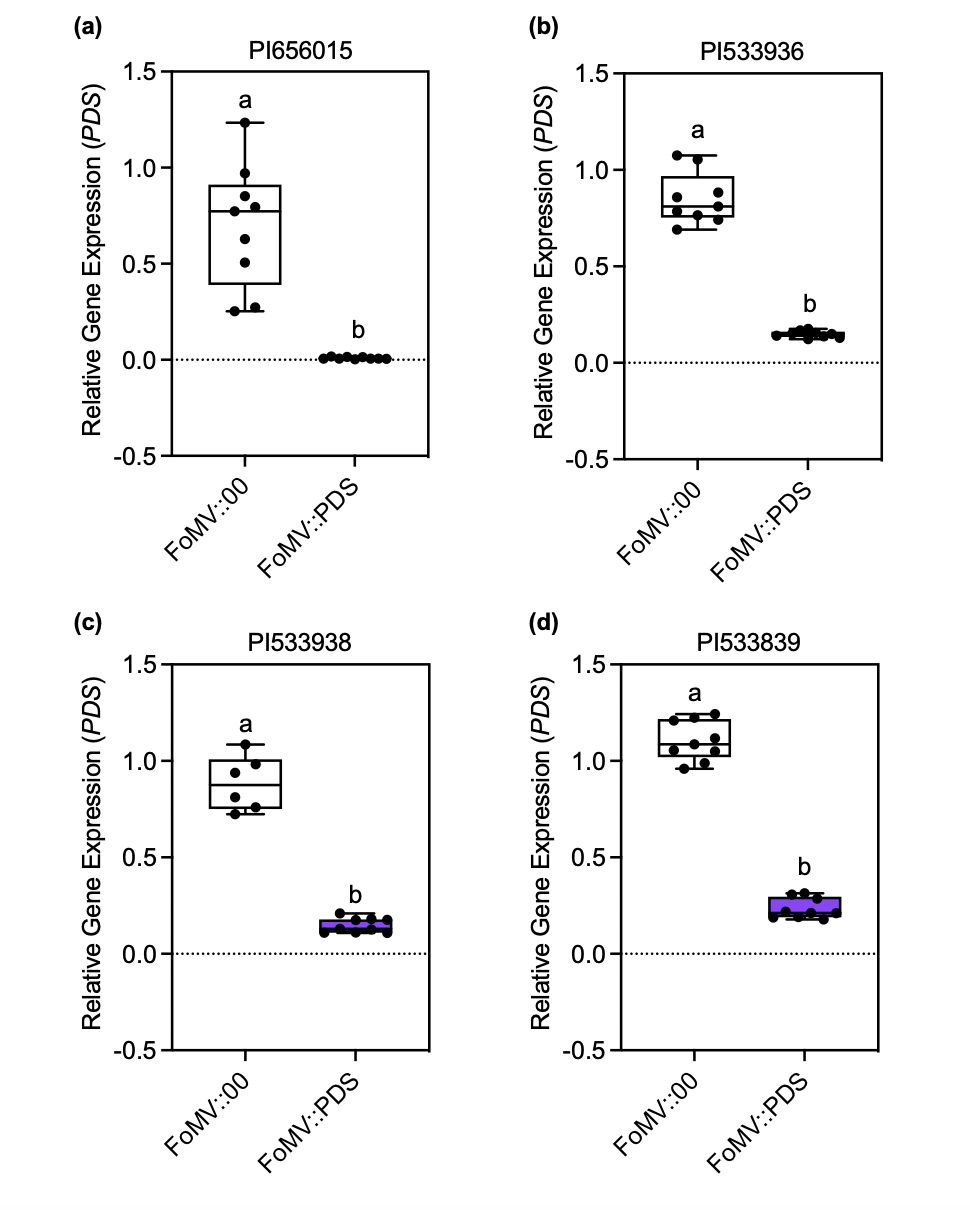


Figure S6. RT-qPCR of *PDS* gene expression in (a) P1656015, (b) PI533936, (c) PI533938, and (d) PI533839 sorghum at 21 dpi (leaf 7). *PDS* gene expression was normalized to *Protein Phosphatase 2A-2* (*PP2A*) gene expression. Data are represented as box plots displaying the 25-75% interquartile range, split by a median line. Whiskers represent maximum and minimum values. Significantly different values (p<0.05) were determined using a one-way ANOVA followed by Tukey’s post hoc test and are represented by different lowercase values. Experiments were conducted three times with similar results.
