# Supplementary material for: Characterization of a foxtail mosaic virus vector for gene silencing and analysis of innate immune responses in Sorghum bicolor
Source: Mol Plant Pathol. 2022 Sep 11;24(1):71–9. doi: 10.1111/mpp.13270 (PMC9742499; doi:10.1111/mpp.13270)
Supplement: Supplementary file 7 — Figure S7 Reverse transcription‐PCR analysis of RLCK gene fragment stability in FoMV. (a) Leaf 6 and (b) leaf 7 were sampled at 21 days postinoculation. Amplicons representing FoMV with no gene inserts migrate to 315 bp. Intact FoMV containing RLCK1 and RLCK2 gene fragments migrate to 614 bp, and FoMV containing the RLCK3 gene fragment migrates to 594 bp. RLCK1/2/3 plants were coinfected with FoMV::RLCK1, FoMV::RLCK2, and FoMV::RLCK3 vectors. Protein Phosphatase 2A‐2 (PP2A) was used as an internal reference control. Experiments were conducted three times with similar results [file MPP-24-71-s008.docx]

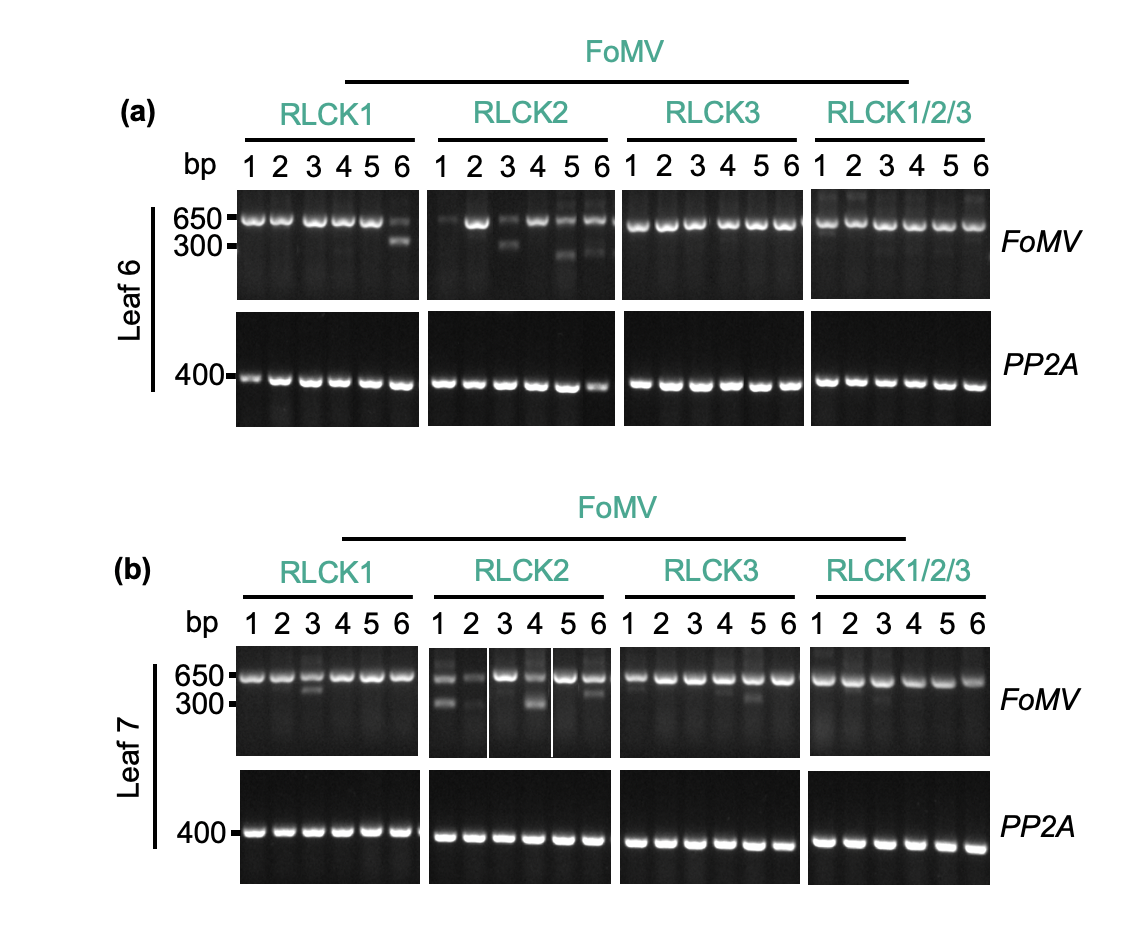


Figure S7. RT-PCR analysis of *RLCK* gene fragment stability in FoMV. (a) Leaf 6 and (b) leaf 7 were sampled at 21 dpi. Amplicons representing FoMV with no gene inserts migrate to 315 bp. Intact FoMV containing *RLCK1* and *RLCK2* gene fragments migrate to 614 bp, and FoMV containing the *RLCK3* gene fragment migrates to 594 bp. RLCK1/2/3 plants were co-infected with FoMV::RLCK1, FoMV::RLCK2, and FoMV::RLCK3 vectors. *Protein Phosphatase 2A-2* (*PP2A*) was used as an internal reference control. Experiments were conducted three times with similar results. White lines indicate where gel images were merged.
