# Supplementary material for: Characterization of a foxtail mosaic virus vector for gene silencing and analysis of innate immune responses in Sorghum bicolor
Source: Mol Plant Pathol. 2022 Sep 11;24(1):71–9. doi: 10.1111/mpp.13270 (PMC9742499; doi:10.1111/mpp.13270)
Supplement: Supplementary file 8 — Figure S8 Reverse transcription‐PCR analysis of RLCK1, RLCK2, and RLCK3 gene fragment inserts in FoMV in BTx623 plants coinfected with all three RLCK gene silencing constructs. An FoMV forward primer was used with gene‐specific reverse primers to differentiate viral constructs (see Table S2). Amplicons for FoMV containing RLCK1, RLCK2, or RLCK3 gene fragments migrate to 279, 304, or 282 bp, respectively. Protein Phosphatase 2A‐2 (PP2A) was used as an internal reference control. The experiment was conducted two times with similar results [file MPP-24-71-s003.docx]

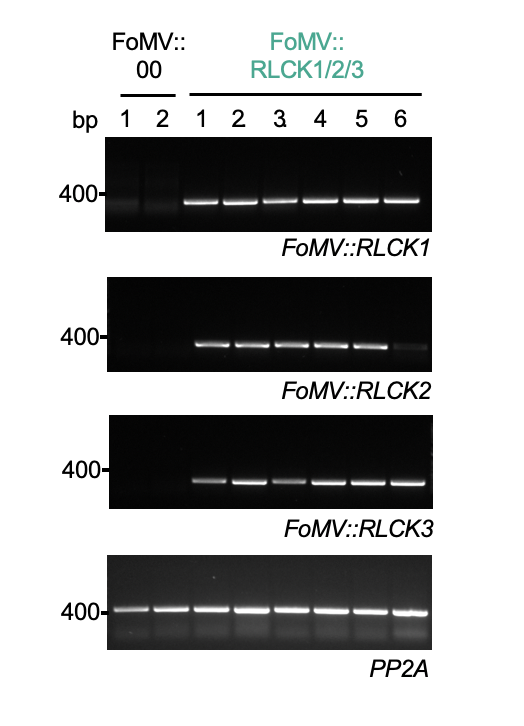


Figure S8. RT-PCR analysis of *RLCK1, RLCK2,* and *RLCK3* gene fragment inserts in FoMV in BTx623 plants co-infected with all three *RLCK* gene silencing constructs. An FoMV forward primer was used with gene specific reverse primers to differentiate viral constructs (See Table S2). Amplicons for FoMV containing *RLCK1, RLCK2,* or *RLCK3* gene fragments migrate to 279 bp, 304 bp, and 282 bp, respectively. *Protein Phosphatase 2A-2* (*PP2A*) was used as an internal reference control. The experiment was conducted two times with similar results.
