# Supplementary material for: Characterization of a foxtail mosaic virus vector for gene silencing and analysis of innate immune responses in Sorghum bicolor
Source: Mol Plant Pathol. 2022 Sep 11;24(1):71–9. doi: 10.1111/mpp.13270 (PMC9742499; doi:10.1111/mpp.13270)
Supplement: Supplementary file 9 — Figure S9 Reverse transcription‐quantitative PCR analysis of endogenous RLCK1, RLCK2, and RLCK3 gene expression levels in mock and FoMV empty vector‐treated plants. Gene expression was calculated relative to Protein Phosphatase 2A‐2 (PP2A). Data are presented as box plots displaying the 25%–75% interquartile range, split by a median line. Whiskers represent maximum and minimum values. Statistical significance (p < 0.05) was determined using a one‐way analysis of variance followed by Tukey’s post hoc test and are denoted by different lowercase letters [file MPP-24-71-s013.docx]

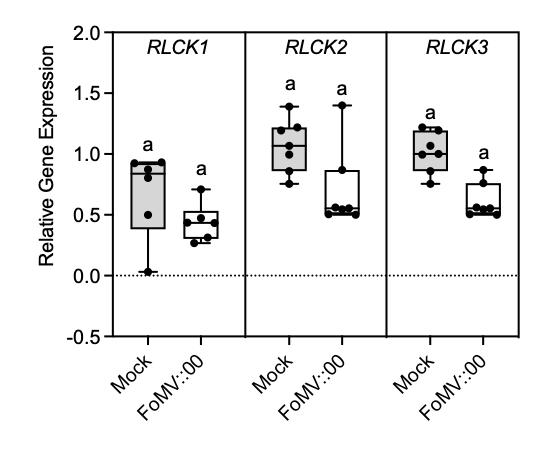


Figure S9. RT-qPCR analysis of endogenous *RLCK1*, *RLCK2,* and *RLCK3* gene expression levels in mock and FoMV empty vector-treated plants. Gene expression was calculated relative to *Protein Phosphatase 2A-2* (*PP2A*). Data are presented as box plots displaying the 25-75% interquartile range, split by a median line. Whiskers represent maximum and minimum values. Statistical significance (p<0.05) was determined using a one-way ANOVA followed by Tukey’s post hoc test and are denoted by different lowercase letters.
