# Supplementary material for: Characterization of a foxtail mosaic virus vector for gene silencing and analysis of innate immune responses in Sorghum bicolor
Source: Mol Plant Pathol. 2022 Sep 11;24(1):71–9. doi: 10.1111/mpp.13270 (PMC9742499; doi:10.1111/mpp.13270)
Supplement: Supplementary file 10 — Table S1 Gene fragments used for FoMV‐induced gene silencing in sorghum [file MPP-24-71-s011.docx]

Table S1. Gene fragments used for FoMV-induced gene silencing in sorghum.

| **Gene** | **Phytozome Accession #** | **Fragment Position (CDS)** | **Orientation** | **Size** |
| --- | --- | --- | --- | --- |
| *PDS* | Sobic.006G232600 | 1306-1618 | Antisense | 312 bp |
| *Ub* | Sobic.010G239500 | 781-1080 | Antisense | 300 bp |
| *RLCK1* | Sobic.001G033400 | 821-1120 | Antisense | 300 bp |
| *RLCK2* | Sobic.001G421300 | 796-1095 | Antisense | 300 bp |
| *RLCK3* | Sobic.009G011700 | 919-1198 | Antisense | 280 bp |
