# Supplementary material for: Characterization of a foxtail mosaic virus vector for gene silencing and analysis of innate immune responses in Sorghum bicolor
Source: Mol Plant Pathol. 2022 Sep 11;24(1):71–9. doi: 10.1111/mpp.13270 (PMC9742499; doi:10.1111/mpp.13270)
Supplement: Supplementary file 11 — Table S2 Primer sequences used for reverse transcription (RT)‐PCR and RT‐quantitative PCR [file MPP-24-71-s012.docx]

Table S2. Primer sequences used for RT-PCR and RT-qPCR.

| **Target** | **Forward Primer** | **Reverse Primer** | **Purpose** |
| --- | --- | --- | --- |
| *FoMV* | 5' -TCTGTACCGTACGATGAGCCC-3' | 5'-GCTGCGTTACTGTTAGGTCG-3' | RT-PCR |
| *FoMV:*  *RLCK1* | 5’-ATAGTCGGCCTCATCTTAGC-3’ | 5'-GCTGCGTTACTGTTAGGTCG-3' | RT-PCR |
| *FoMV:*  *RLCK2* | 5’-GTCACAACCTGATCCATGCC-3’ | 5'-GCTGCGTTACTGTTAGGTCG-3' | RT-PCR |
| *FoMV:*  *RLCK3* | 5’-GCTGTTACCACCTCGTCCAT-3’ | 5'-GCTGCGTTACTGTTAGGTCG-3' | RT-PCR |
| *PDS* | 5'-CCAGAAACTTTGCCAGCACC-3' | 5'-CGATCAGGAACACCCTGCTT-3' | RT-qPCR |
| *Ub* | 5’-TGGAGGTGGAGTCTTCTGATAC-3’ | 5’-TGGATGTTGTAGTCTGCCAAAG-3’ | RT-qPCR |
| *RLCK1* | 5'-GCTGATTGGATACTGCGTAGAG-3' | 5'-GGAAGTATGAGCCTCTCCTAAAC-3' | RT-qPCR |
| *RLCK2* | 5'-AAGAAGCTCAAGCTCGACAG-3' | 5'-CTCCAAGCAGTAGCCGATAAG-3' | RT-qPCR |
| *RLCK3* | 5'-ACTGCCTTGAAGACGAACAG-3' | 5'-CAGGACAGTGGCTGGAAATAG-3' | RT-qPCR |
| *PP2A* | 5'-CTTGATCGCATACAGGAGGTTC-3' | 5'-CGGATGAGAGGAGCATCAACA-3' | RT-PCR |
| *PP2A* | 5'-AACCCGCAAAACCCCAGACTA-3' | 5'-TACAGGTCGGGCTCATGGAAC-3' | RT-qPCR |
