# Supplementary material for: Characterization of a foxtail mosaic virus vector for gene silencing and analysis of innate immune responses in Sorghum bicolor
Source: Mol Plant Pathol. 2022 Sep 11;24(1):71–9. doi: 10.1111/mpp.13270 (PMC9742499; doi:10.1111/mpp.13270)
Supplement: Supplementary file 12 — Table S3 Nucleic acid identity of RLCK1, RLCK2, and RLCK3 gene fragments used for FoMV virus‐induced gene silencing [file MPP-24-71-s007.docx]

Table S3. Nucleic acid identity of RLCK1, RLCK2, and RLCK3 gene fragments used for FoMV VIGS.

|  | **FoMV::RLCK1** | **FoMV::RLCK2** | **FoMV::RLCK3** |
| --- | --- | --- | --- |
| *SbRLCK1* | 100% | 64% | 67% |
| *SbRLCK2* | 34% | 100% | 45% |
| *SbRLCK3* | 41% | 72% | 100% |
