# Supplementary material for: Characterization of a foxtail mosaic virus vector for gene silencing and analysis of innate immune responses in Sorghum bicolor
Source: Mol Plant Pathol. 2022 Sep 11;24(1):71–9. doi: 10.1111/mpp.13270 (PMC9742499; doi:10.1111/mpp.13270)
Supplement: Supplementary file 13 — File S1 Supplemental experimental procedures. Viral vector construction, viral inoculum preparation and sorghum inoculation, FoMV‐induced gene silencing, RT‐PCR and RT‐qPCR analysis, immune assays, supplemental references [file MPP-24-71-s004.docx]

# Supplemental Experimental Procedures

## Viral Vector Construction

A DNA-based FoMV infectious clone previously developed for gene silencing and overexpression in maize [(Mei et al., 2016; Mei et al., 2019)](https://paperpile.com/c/497bZq/YM8H+6Q6J) was modified for VIGS in sorghum. Approximately 300 bp fragments from the 5’ end of *PDS*, *Ub,* *RLCK1*, *RLCK2*, and *RLCK3* coding sequences were selected for gene silencing using the Sol Genomics Network VIGS Tool (<https://vigs.solgenomics.net/>) (Table S1). The antisense sequence of gene fragments were synthesized as double stranded DNA sequences (gBlocks^TM)^ (Integrated DNA Technologies) with 30 bp overhangs complementary to MCSI of FoMV for seamless cloning using NEBuilder HiFi DNA Assembly (New England BioLabs). FoMV backbones were digested at MCSI using the MluI restriction enzyme, dephosphorylated, and used for HiFi DNA Assembly, according to manufacturer instructions. All constructs were confirmed by Sanger sequencing (Iowa State University DNA Facility) before conducting VIGS experiments. The details of gene fragments used for FoMV-induced gene silencing for the five genes tested in this study are shown in Table S1.

## Viral Inoculum Preparation and Sorghum Inoculation

Inoculum for VIGS experiments was generated using *Nicotiana benthamiana.* Seeds were germinated on LC1 Grower’s Mix (Sungro) in a growth room maintained at 24°C with a 16 h photoperiod (185 photosynthetically active radiation (PAR)), with no humidity control. All plants were fertilized weekly with Peter’s Excel 15-5-15 (ICL Performance Products) at 300 parts per million (ppm). Viral inoculum was prepared by syringe infiltrating 4-week-old *N. benthamiana* with *Agrobacterium tumefaciens s*train GV3101 containing FoMV vectors resuspended in infiltration buffer containing 10 mM MgCl_2_,10 mM MES, and 200 mM acetosyringone (pH 5.6) and diluted to OD_600_=0.1, as described previously [(Mei et al., 2019)](https://paperpile.com/c/497bZq/6Q6J). Leaf tissue was collected 7 days post-infiltration, lyophilized, and stored at -20 °C until use. The presence of target gene fragments in all inoculum was confirmed by reverse-transcription (RT)-PCR and Sanger sequencing (Iowa State University DNA Facility) before use. At 7 days post-germination, sorghum seedlings (2-3 leaf stage, depending on the genotype) were mechanically inoculated by dusting with 600-mesh carborundum and rubbing with the sap of *N. benthamiana* leaf tissue containing FoMV viral particles. Prior to inoculation, lyophilized *N. benthamiana* tissue was homogenized in a 50 mM potassium phosphate buffer (pH 7.0) at a 1:20 (w:v) ratio.

## FoMV-Induced Gene Silencing

Six sorghum genotypes (RTx430, BTx623, PI656015, PI533936, PI533839 and PI533938) were chosen for assessment of FoMV-induced gene silencing. RTx430 was chosen because it is transformable, has been largely utilized by the sorghum research community, and the availability of optimized transformation protocols [(Liu and Godwin, 2012)](https://paperpile.com/c/497bZq/OBcoH). The other five genotypes, including the reference line BTx623, were included because they are part of the sorghum association panel [(Casa et al., 2008)](https://paperpile.com/c/497bZq/ZhS1s). Seeds of each sorghum genotype were germinated in LC1 Grower’s Mix (Sungro) and maintained in a greenhouse at 25-28 °C, supplemented with 16 h of light (185 photosynthetically active radiation (PAR)) and 47-55% relative humidity.

## RT-PCR and qRT-PCR Analysis

RNA was extracted from sorghum plants 14-28 dpi, as specified in figures, using Trizol reagent (Thermo Fisher Scientific, Waltham, MA, USA) and normalized prior to cDNA synthesis using the Maxima cDNA Synthesis Kit with DNase (Thermo Fisher Scientific, Waltham, MA, USA). FoMV was detected by RT-PCR using primers designed to span MCSI in order to assess fragment insert stability. *Protein Phosphatase 2A-2* (*PP2A)* was used as an internal reference control [(Sudhakar Reddy et al., 2016)](https://paperpile.com/c/497bZq/9Is1j). Primers were designed to flank MCSI in order to monitor the stability of gene silencing insertions. VIGS was assessed using the iTaq Universal SYBR Green Supermix kit (BioRad, Hercules, CA, USA). Primers used to assess *PDS*, *Ub,* *RLCK1*, *RLCK2, RLCK3*, and *PP2A* expression are specified in Table S2. RT-qPCR assays were run on a BioRad CFX96 Real-Time PCR detection system (BioRad, Hercules, CA, USA), and analyzed using the 2^(-Delta Delta CT)^ method to determine gene expression levels relative to *PP2A* [(Livak and Schmittgen, 2001)](https://paperpile.com/c/497bZq/MgwBe). Only samples determined to have intact VIGS inserts carried by FoMV from RT-PCR were included in the RT-qPCR analysis.

## Immune Assays

Oxidative burst assays were adapted from a previously established protocol for Arabidopsis [(Bredow et al., 2019)](https://paperpile.com/c/497bZq/cMvPg). Briefly, leaf discs were collected from sorghum plants of respective treatments 21 dpi and placed in 96-well plates containing 100 µL of water with the adaxial side facing upward. The next day, water was replaced with 100 µL of elicitor solution containing 1 µM of the luminol derivative L-012 (Wako Chemicals, St. Louis, MO), 10 µg/mL of horseradish peroxidase (HRP) (Sigma Aldrich, St. Louis, MO), and either 1 µM of flg22 (VWR, Radnor, PA) or 10 µg/mL of chitin (Sigma Aldrich, St. Louis, MO). Luminescence measurements were taken on a Promega GloMax plate reader every 2 min for 40 min with a 1 sec integration time.

For infection assays, *P. syringae pv. syringae* (B728a) was grown in King’s B (KB) broth containing 50 µg/mL of rifampicin overnight at 28 °C. The next day cultures were resuspended in phosphate buffer (pH 7.5) and diluted to OD_600_=0.2 (1 x 10^8^ colony forming units (CFU)/mL) with 0.04% Silwet L-77 (Thermo Fisher Scientific, Waltham, MA) added immediately before infections. At 21 days post inoculation with FoMV viral vectors, leaf 7 of BTx623 plants were sprayed on the adaxial and abaxial surfaces until evenly wet. Sprayed leaves were placed in plastic bags for 24 h to increase humidity. Three days after bacterial infections, leaf discs were collected from a total of 8 plants per treatment and fully homogenized in phosphate buffer (pH 7.5) and plated on KB plates containing 50 µg/mL of rifampicin. CFUs were counted 2-3 days after plating.

For *X. vasicola pv. holcicola* (Mex-1) infections, bacteria was grown in Nutrient Broth (NB) overnight at 28 °C. Cultures were pelleted and resuspended in 10 mM MgCl_2_ to 2 x 10^7^ CFU/mL (OD_600_=0.01). For lesion measurements, 100 µL of bacterial suspension was infiltrated into the abaxial surface leaf 6 of BTx623 plants 21 days after inoculation with FoMV, and measured 6 days after infiltration. For CFU measurements, leaf 7 was infiltrated with 1 mL of bacterial suspensions and colony counts were taken from a total of 6 plants per treatment at 24 hours post infiltration as described above.

## Supplemental References

[Bredow, M., Sementchoukova, I., Siegel, K. & Monaghan, J. (2019) Pattern-Triggered Oxidative Burst and Seedling Growth Inhibition Assays in Arabidopsis thaliana. *Journal of visualized experiments*, e59437, doi:10.3791/59437.](http://paperpile.com/b/497bZq/cMvPg)

[Casa, A.M., Pressoir, G., Brown, P.J., Mitchell, S.E., Rooney, W.L., Tuinstra, M.R., et al. (2008) Community Resources and Strategies for Association Mapping in Sorghum. *Crop science*, 48, 30–40.](http://paperpile.com/b/497bZq/ZhS1s)

[Liu, G. & Godwin, I.D. (2012) Highly efficient sorghum transformation. *Plant cell reports*, 31, 999–1007.](http://paperpile.com/b/497bZq/OBcoH)

[Livak, K.J. & Schmittgen, T.D. (2001) Analysis of Relative Gene Expression Data Using Real-Time Quantitative PCR and the 2−ΔΔCT Method. *Methods*, 25, 402–408.](http://paperpile.com/b/497bZq/MgwBe)

[Mei, Y., Beernink, B.M., Ellison, E.E., Konečná, E., Neelakandan, A.K., Voytas, D.F., et al. (2019) Protein expression and gene editing in monocots using foxtail mosaic virus vectors. *Plant direct*, 3, e00181.](http://paperpile.com/b/497bZq/6Q6J)

[Mei, Y., Zhang, C., Kernodle, B.M., Hill, J.H. & Whitham, S.A. (2016) A Foxtail mosaic virus Vector for Virus-Induced Gene Silencing in Maize. *Plant physiology*, 171, 760–772.](http://paperpile.com/b/497bZq/YM8H)

[Sudhakar Reddy, P., Srinivas Reddy, D., Sivasakthi, K., Bhatnagar-Mathur, P., Vadez, V. & Sharma, K.K. (2016) Evaluation of Sorghum [*Sorghum bicolor* (L.)] Reference Genes in Various Tissues and under Abiotic Stress Conditions for Quantitative Real-Time PCR Data Normalization. *Frontiers in plant science*, 7, 529.](http://paperpile.com/b/497bZq/9Is1j)
